# Comprehensive profiling of accessible surface glycans of mammalian sperm using a lectin microarray

**DOI:** 10.1186/1559-0275-11-10

**Published:** 2014-03-16

**Authors:** Ai-Jie Xin, Li Cheng, Hua Diao, Peng Wang, Yi-Hua Gu, Bin Wu, Yan-Cheng Wu, Guo-Wu Chen, Shu-Min Zhou, Shu-Juan Guo, Hui-Juan Shi, Sheng-Ce Tao

**Affiliations:** 1 Shanghai Center for Systems Biomedicine, Key Laboratory of Systems Biomedicine (Ministry of Education), Shanghai Jiao Tong University, Shanghai 200240, China; 2 State Key Laboratory of Oncogenes and Related Genes, Shanghai 200240, China; 3 School of Biomedical Engineering, Shanghai Jiao Tong University, Shanghai 200240, China; 4 Shanghai Jiai Genetics & IVF Institute, Obstetrics and Gynecology Hospital of Fudan University, Shanghai Key Laboratory of Female Reproductive Endocrine-Related Diseases, Shanghai 200011, China; 5 China National Population and Family Planning Key Laboratory of Contraceptive Drugs and Devices, SIPPR, Shanghai 200032, China; 6 State Key Laboratory of Bioreactor Engineering, East China University of Science and Technology, Shanghai 20037, China

**Keywords:** Sperm, Glycocalyx, Surface glycan, Lectin microarray

## Abstract

It is well known that cell surface glycans or glycocalyx play important roles in sperm motility, maturation and fertilization. A comprehensive profile of the sperm surface glycans will greatly facilitate both basic research (sperm glycobiology) and clinical studies, such as diagnostics of infertility. As a group of natural glycan binders, lectin is an ideal tool for cell surface glycan profiling. However, because of the lack of effective technology, only a few lectins have been tested for lectin-sperm binding profiles. To address this challenge, we have developed a procedure for high-throughput probing of mammalian sperm with 91 lectins on lectin microarrays. Normal sperm from human, boar, bull, goat and rabbit were collected and analyzed on the lectin microarrays. Positive bindings of a set of ~50 lectins were observed for all the sperm of 5 species, which indicated a wide range of glycans are on the surface of mammalian sperm. Species specific lectin bindings were also observed. Clustering analysis revealed that the distances of the five species according to the lectin binding profiles are consistent with that of the genome sequence based phylogenetic tree except for rabbit. The procedure that we established in this study could be generally applicable for sperm from other species or defect sperm from the same species. We believe the lectin binding profiles of the mammalian sperm that we established in this study are valuable for both basic research and clinical studies.

## Introduction

The membrane surface of sperm is coated with a thick layer of glycans, *i.e.*, the sperm glycocalyx, which is the first interface between sperm and the environment [1]. For example, the thickness of sperm glycocalyx of guinea boar varies from 20–60 nm on different sections of sperm [2]. It is estimated that sperm glycocalyx is comprised of 50 to 150 different glycoconjugates, and these glycoconjugates distribute heterogeneously from head to tail of sperm [3]. Although the roles of sperm glycocalyx have not been fully illustrated, it is well known that glycans play important roles in sperm maturation, sperm protection during transportation in female reproductive tract, acrosome reaction, and the final sperm-egg recognition/fertilization [1,3]. For example, glycosylation plays a critical role during epididymal sperm maturation. The increase in the abundance of sialic acid on sperm surface is a main indication of sperm maturation, which is also the major contributor of the negative charge of sperm [4].

Several techniques could be applied for deciphering both the composition and structure of complex glycan samples. Among these, the most powerful one is mass spectrometry, which has already been frequently applied in glycobiology [5,6]. Mass spectrometry has already been successfully applied for the analysis of glycans on the cell membrane [7-10]. However, the sophistication of the membrane glycan enrichment procedure and the mass spectrometry analysis itself make it not easy for wide applications. As a group of naturally existing glycanbinders, lectins have been used for sperm analysis for many years [11,12]. For example, the binding of sialic acid-specific lectins to the sperm surface of several primate species have already been demonstrated, such as SNA and MALII [13,14]. In addition, positive staining of WGA, TGP and PNA was observed on the membrane of mature spermotozoa, which indicate the existing of NAc-glucosaminylated, fucosylated, and NAc-galactosamine glycan residues, respectively [15]. For lectin based analysis, enzyme, biotin or fluorescent dye conjugated lectin was individually applied for staining and the binding results were readout by microscope or flow-cytometry [16], thus, the basic methodologies for lectin based analysis are immunocytochemistry, immunohistochemistry and flow cytometry, which are labor-intensive, time-consuming, and often require cumbersome sample pre-treatment. Furthermore, these methodologies are usually carried out in a one by one fashion, which is not suitable for global and high-throughput analysis.

To overcome the drawbacks of the traditional lectin based methodologies, combined with the powerful characteristics of microarray format: high-throughput, parallel analysis and miniaturized format, we and others have developed a variety types of lectin microarrays carrying a few to ~100 lectins [17-21]. These lectin microarrays were fabricated on several different functionalized substrate surfaces, such as aldehyde, epbully and NHS derivatized hydrogel. The lectin microarrays have been applied for fast and high-throughput glycan profiling and comparison for a variety of samples, *e.g.*, glycoprotein [22-26], cell lysates [27-29], clinical specimens [30,31], mammalian cells [20,32-34], bacteria [17,35] and virus [36]. Using the lectin microarray, the disease specific or cell type specific lectins could be easily identified. Coupling this results with other techniques, e.g., mass spectrometry, the biologically or clinically important glycans or glycoproteins could be further digged out [37].

To obtain a comprehensive profile of the accessible glycans on mammalian sperm surface, we have developed a procedure for high-throughput probing of mammalian sperm on a lectin microarray with 91 lectins. Normal sperm from human, sheep, boar, bovine and rabbit were collected and analyzed on the lectin microarray. Positive bindings of a set of ~50 lectins were observed for all the sperm, which indicate a wide range of glycans are on the surface of mammalian sperm. Species specific lectin bindings were also observed. Clustering analysis revealed that the distances of the five species according to the lectin binding profiles are consistent with that of the genome sequence based phylogenetic tree except for rabbit. The procedure that we established in this study could be generally applicable for the analysis of sperm from other species or defect sperm from the same species.

## Materials and methods

### Sperm preparation

All procedures were approved by the Ethics Committee of Shanghai Institutes for Biological Sciences Chinese Academy of Sciences. The samples used in this study were ejaculated sperm from human, boar and bull, and epididymal cauda sperm from goat and rabbit. Human semen with normal sperm count (sperm count ≥ 15 × 10^6^/ml) and normal motility (progressive motility (PR) ≥ 40%) were collected from Shanghai Jiai Genetics & IVF Institute and washed twice in phosphate buffered saline (PBS, pH 7.2) after liquefaction 30 min, by centrifugation with 500 g for 5 min. Semen from three Changbai Mountain boars were also washed with the same procedure as that of human sperm. Frozen semen samples of Hostein bull were bought from SEMEX (Shanghai, China). They were thawed by immersion in a 38°C water bath for 10 s and then washed twice as that of human sperm. Sperm of Guanzhong dairy goats and New Zealand white rabbits were released from cauda epididymis into PBS for about 10 min and collected by centrifugation. All the washed sperm samples were fixed with 2% paraformaldehyde/0.2% glutaraldehyde for 30 min and washed twice with PBS, stored at 4°C.

### Lectin microarray fabrication

Lectin microarray was prepared as previously described [20]. Briefly, ninety-one lectins obtained from EY Laboratories (San Mateo, CA) and Vector Laboratories (Burlingame, CA) which were dissolved in spotting solution (PBS containing 0.02% Tween-20, 25% glycerol and 0.05 μg/μl bovine serum albumin (BSA)) at a final concentration of 1 μg/μl and printed on OPPolymer Slide H slides (CapitalBio, Beijing, China) by a SmartArray™ -48 microarrayer (CapitalBio, Beijing, China). Each lectin was printed in triplicate per block with 18 × 16 arrangement and 12 blocks were printed per slide. After printing, the slides were incubated at 4°C overnight to ensure a maximum protein coupling on the surface and stored at 4°C.

### Probing sperm on the lectin microarray

Lectin microarrays were blocked in 10 mM Tris Buffered Saline with 0.5% (v/v) Tween-20 (TBST) for 1 h at room temperature and washed three times (once in PBST, twice in PBS). Meanwhile, the fixed sperm were counted with Makler counting chambers (Irvine Scientific, Santa Ana, CA, USA) to determine the concentrations, sperm were labeled with propidine iodide (PI, 20 μg/ml) in PBS buffer for 20 min. The PI-labeled sperm were collected with centrifugation and re-suspended in binding buffer (PBS with 50 μM CaCl_2_ and 50 μM MnCl_2_). Five million spermatozoa in a volume of 200 μl was loaded per block, the lectin microarray was then incubated at room temperature for 1 h. The probing of each sperm sample was repeated for fourblocks. After incubation, the lectin microarrays were submerged in PBST and inverted gently to remove the excess and/or unbound sperm. After the microarrays were air-dried, the results were recorded by microarray scanner (GenePix 4200A, Molecular Devices, Sunnyvale, CA) at 5 μm resolution. The scanning condition was set to 532 nm filter and 40% PMT value.

### Lectin microarray data analysis

The lectin-sperm binding intensity (F532) and the local background intensity (B532) were extracted by GenePix pro 6.0 (Molecular Devices, Sunnyvale, CA). The signal to backgroundratios (S/B) (F532/B532) was calculated for all the lectins on the microarray. The S/B of the 12 replicate spots for each lectin was averaged and the standard deviation of each lectin was also calculated. The PI labeling efficiency of each sperm sample was measured by a flurorescence spectrometry, the microarray results were then normalized against the labeling efficiencies to reduce the effect of labeling variation.

To call the positive lectin binding, the cut off was set as S/B ≥ 2. All statistic anlysis was conducted by SPSS16.0.

### Clustering

The hierarchically clustered heatmap was created by using R-3.0.1 (http://www.r-project.org/). The lectin-sperm binding data of the lectins which showed positive binding (S/B ≥ 2) to at least one sperm sample were used as input for clustering. Lectins and species were clustered using the euclidean distance metric. Green represents strong binding between the lectins and the sperm samples while red represents a weak binding.

## Results

### Schematic diagram

**Figure 1:**
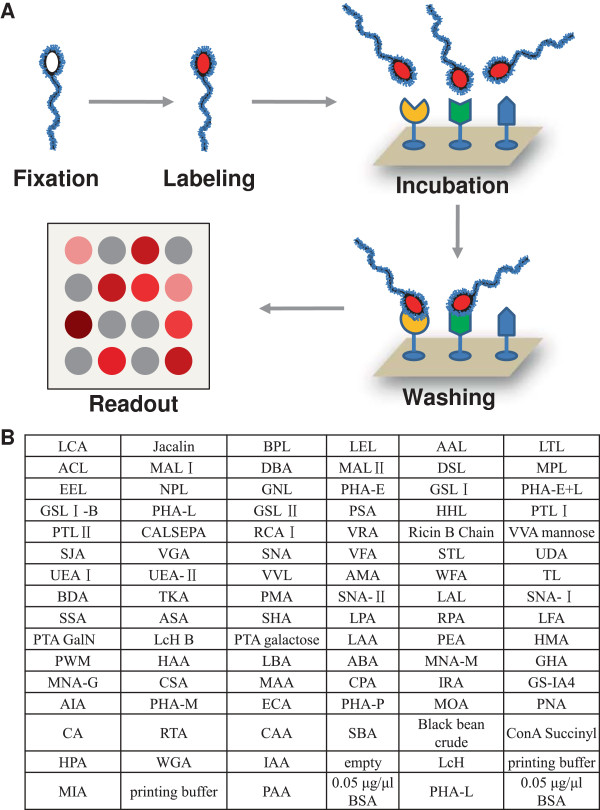
**The schematic and workflow of profiling sperm surface glycans on lectin microarray. (A)**. The procedure of analysis sperm on lectin microarray is similar to that of other cells except a fixation step before label the sperm with a fluorescent dye. A typical workflow is composed of five steps, i.e., fixation, labeling, incubation, washing and the final readout. **(B)**. The layout the lectin microarray with 91 lectins, each lectin was repeated for three spots on one block.

The schematic of probing sperm on lectin microarray is similar to that of probing other intact cells [20] (Figure 1A). The differences lie in the sample pre-treatment and labeling. To get reliable comparison among the sperm from different species and also to reduce the effect of the fast motility of live sperm on sperm-lectin binding, a good practice is to keep the integrity and the surface glycans of the sperm through fixation. By fixation right after the collection of each sperm sample, we can collect sperm from different animals, time and location, and compare them on a single microarray or a single experiment. Thus, in addition to traditional steps for probing intact cells on lectin microarray, *i.e.*, labeling, binding, washing and readout, a fixation step is included for sperm analysis. To control the quality of the microarray probing, lectin printing buffer and BSA were also included on the lectin microarray as negative controls, the layout the lectin microarray was shown in Figure 1B.

### Sample preparation for lectin microarray analysis

To prepare sperm for lectin microarray analysis, initially, we collected sperm samples from healthy human, model organisms (*i.e.*, mouse, rat and rabbit) and livestock (*i.e.*, boar, bull and goat). Human reproduction is always one of the most important research field, and the sperm glycosylation may play a key role for this. As for model organisms, the profile of the sperm surface glycans could serve as a valuable resource for related biological studies. As for livestocks such as boar, bull and goat, the study of the sperm surface glycans may provide useful information for the improvement of the quality of these animals during breeding. However, the size and morphology of the sperm of these species are quite diverse, for example, the length of the sperm of human, boar, bull, goat and rabbit is about 50–70 μm, while the length of mouse sperm is about 150 μm, and the length of rat sperm is even longer [38]. Moreover, the head of the sperm of human, boar, bull, goat and rabbit is round, while that of mouse and rat is sickle shape. Under the same condition and procedure that we have established, the analysis of the sperm of mouse and rat on the lectin microarray was not successful, especially rat sperm. These failures may due to the longer size and complex morphology of these sperm compared to human sperm. Thus we focused on analyzing the sperm of human, boar, bull, goat and rabbit in this study.

### Analysis of the mammalian sperm on the lectin microarray

To get the lectin binding profiles of the mammalian sperm. The fixed sperm were labeled with propidine iodide, which is nucleic acid specific. Once the dye is bound to nucleic acid, the fluorescence excitation maximum is 535 nm and the emission maximum is 617 nm, and its fluorescent signal is enhanced 20- to 30-fold, thus, the washing step is not necessary after staining. The labeled sperm of different species were then incubated on a lectin microarray with 91 lectins individually. There were twelve identical blocks on a lectin microarray, to assure the reliability of the microarray results, the probing of sperm sample of each species was repeated in four blocks. After washing and drying, the signals of the lectin microarrays were recorded by a microarray scanner. S/B were calculated for all the lectins. To improve the reliability of the sperm profiling, sperm samples from 10 healthy men, and sperm samples from 3 animals of other 5 five species were prepared. To reduce the sample-to-sample variation, the sperm samples of the same species were mixed together and probed on the lectin microarray. To improve the reliability, each sperm sample was technically repeated for 4 blocks on a single lectin microarray. The variations of the sperm of each species were then calculated based on the replicates.

**Figure 2:**
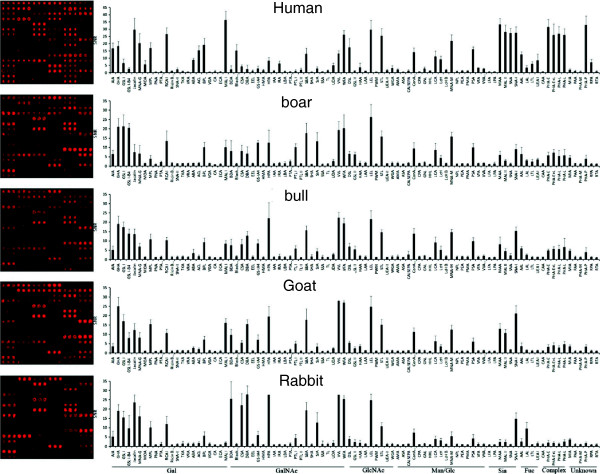
**The results of analyzed sperm of five mammalian species on the lectin microarray.** For all the five species that have been analyzed, representative images were shown on the left. Quantitative results were also shown on the right. For a given lectin, the error bar was the standard deviation of 12 duplicated lectin spots from two repeated microarray experiments.

The lectin binding patterns of sperm from human, boar, bull, goat and rabbit were shown in Figure 2. Using S/B > =2 as cutoff, the number of lectins with positive binding to the sperm of human, boar, bull, goat and rabbit were 53, 46, 44, 42 and 37, respectively. These lectins covers a wide range of glycan specificity, for example, galactose (Gal), N-acetylgalactosamine (GalNAc), N-acetylglucosamine (GlcNAc), mannose/glucose (Man/Glc), sialic acids (Sia), fucose (Fuc) and complex-type glycan.

For example, human sperm bound to 14 Gal specific lectins (AIA, GHA, GSL I, GSL I-B4, Jacalin, MNA-G, MOA, MPL, RCA I, SNA-II, ABA, ACL, BPL, MAL I), 12 GalNAc specific lectins (BDA, Black bean crude, CSA, DBA, GS-IA4, HPA, IRA, SBA, SJA, UDA, VVL, WFA), 5 GlcNAc specific lectins (DSL, HAA, LEL, STL, WGA), 9 Man/Glc specific lectins (AMA, ConA Succinyl, GNL, LCA, LcH, MNA-M, PSA, VFA, VVA mannose), 4 Sia specific lectins (MAA, MAL II, SNA, SNA-I), 4 Fuc specific lectins (AAL, LAL, LTL, UEA I), 3 complex-type glycan specific lectins (PHA-E, PHA-E + L, PHA-L), and 2 lectins (PHA-P, RPA) with unknown glycan specificity on the lectin microarray. Boar sperm bound to 11 Gal-binders (AIA, GHA, GSL I, GSL I-B4, Jacalin, MNA-G, MPL, PTA galactose, RCA I, BPL, MAL I), 13 GalNAc-binders (BDA, CSA, DBA, GS-IA4, HPA, PTA GalNAc, PTL I, SBA, SJA, UDA, VVL, WFA), 4 GlcNAc-binders (DSL, GSL II, LEL, STL), 6 Man/Glc-binders (ConA Succinyl, GNL, LCA, LcH, MNA-M, PSA), 3 Sia-binders (MAA, MAL II, SNA-I), 4 Fuc-binders (AAL, LAL, LTL, UEA I), 3 complex-binders (PHA-E, PHA-E + L, PHA-L), 2 lectins (MIA, PHA-P) with unknown glycan specificity. Bull spermatozoa bound to 11 Gal-binders (AIA, GHA, GSL I, GSL I-B4, Jacalin, MNA-G, MPL, RCA I, ABA, BPL, MAL I), 11 GalNAc-binders (BDA, CSA, DBA, GS-IA4, HPA, PTL I, SBA, SJA, UDA, VVL, WFA), 4 GlcNAc-binders (DSL, GSL II, LEL, STL), 7 Man/Glc-binders (CALSEPA, ConA Succinyl, GNL, LCA, LcH, MNA-M, PSA), 4 Sia-binders (MAA, MAL II, SNA, SNA-I), 2 Fuc-binders (AAL, LAL), 3 complex-binders (PHA-E, PHA-E + L, PHA-L), 2 lectins (MIA, PHA-P) with unknown glycan specificity. Goat sperm bound to 12 Gal-binders (AIA, GHA, GSL I, GSL I-B4, Jacalin, MNA-G, MPL, RCA I, ABA, ACL, BPL, MAL I), 11 GalNAc-binders (BDA, CSA, DBA, GS-IA4, HPA, PTL I, SBA, SJA, UDA, VVL, WFA), 5 GlcNAc-binders (DSL, GSL II, HAA, LEL, STL), 5 Man/Glc-binders (ConA Succinyl, LCA, LcH, MNA-M, PSA), 3 Sia-binders (MAA, MAL II, SNA-I), 1 Fuc-binder (AAL), 3 complex-binders (PHA-E, PHA-E + L, PHA-L), 2 lectins (MIA, PHA-P) with unknown glycan specificity. Rabbit sperm bound to 10 Gal-binders (AIA, GHA, GSL I, GSL I-B4, Jacalin, MNA-G, MPL, RCA I, BPL, MAL I), 10 GalNAc-binders (BDA, CSA, DBA, GS-IA4, HPA, PTL I, SBA, SJA, VVL, WFA), 4 GlcNAc-binders (DSL, GSL II, LEL, STL), 5 Man/Glc-binders (ConA Succinyl, LCA, LcH, MNA-M, PSA), 3 Sia-binders (MAA, MAL II, SNA-I), 2 Fuc-binder (AAL, LAL), 2 complex-binders (PHA-E, PHA-L), and one lectin (MIA) with unknown glycan specificity.

As shown in Figure 2, both the number of positive lectins and the overall binding intensity of the human sperm were the highest. This may indicates the existing of the most abundant glycans and the most complex glycan structures on the surface of human sperm than that of sperm from other animals that we have tested.

Interestingly, there were also lectins that specifically bound to or not to a given species. For human, the specific lectins with positive bindings were Black bean crude, IRA, WGA, AMA, VFA, VVA mannose and RPA, and the lectins with negative bindings were PTL I, GSL I, and MIA; For boar, the specific lectins with positive bindings were PTA galactose, PTA GalNAc; For bull, the specific lectins with positive bindings were CALSEPA AND LFA; For goat, there was positive lectin ACL and one negative LAL; And for rabbit, there were three negative lectins, *i.e.*, PHA-E + L, PHA-L and PHA-P, all these three lectins are specific for complex and unknown glycan structures, which indicates the missing of those glycan structures on the surface of the rabbit sperm.

### Quantitative analysis of the sperm binding on the lectin microarray

To take the signal intensity into account, the sperm-lectin binding intensity were divided into 4 grades, *i.e.*, strong binding (+++, S/B ≥ 20), medium binding (++, 20 > S/B ≥ 10), weak binding (+, 10 > S/B ≥ 2) and negative binding (-, S/B < 2) (Additional file 1). According to these grades, for human, there were 16, 13, 24 and 37 lectins of strong, medium, weak and negative bindings, respectively; for boar, there were 5, 11, 29 and 45 lectins of strong, medium, weak and negative bindings, respectively; for bull, there were 3, 13, 28 and 46 lectins of strong, medium, weak and negative bindings, respectively; for goat, there were 5, 13, 24 and 48 lectins of strong, medium, weak and negative bindings, respectively; and for rabbit, there were 8, 9, 20 and 54 lectins of strong, medium, weak and negative bindings, respectively. The lectins of all the four grades distributed evenly among the lectin categories of different glycan specificity. This indicates that the overall sperm surface glycans are similar or at least comparable among the five mammalian species.

### The cluster of the five mammalian sperm according to their lectin binding profile is consistent the gene based phylogenetic tree

**Figure 3:**
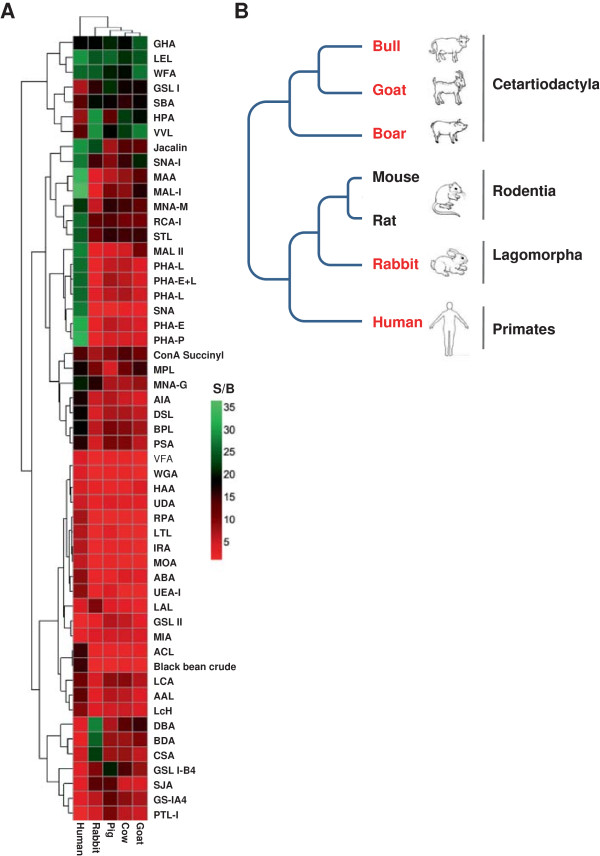
**The clustering of the five species according to the lectin-sperm binding profiles is consistent to the phylogenetic tree. (A)**. The clustered heatmap of the lectin-sperm binding profiles of the five species generated by using the lectins which showed positive bindings (S/B ≥ 2). **(B)**. The phylogenetic tree of the five species (red labeled) modified from Kriegs JO et al. [39] and Murphy WJ et al. [40].

To intuitively compare the difference of lectin-binding profiles among the five mammalian sperm, a clustered heat map was generated (Figure 3A). Obviously, four major clusters were prominent. The first cluster demonstrated strong binding among all the five mammalian sperm consisted of Gal-binder (GHA), GlcNAc-binder (LEL) and GalNAc-binder (WFA). The second cluster with strong binding in boar, bull, goat, rabbit, but weak in human, composed of Gal-binder GSL I and and GalNAc-binders (SBA, HPA and VVL). The third cluster exhibited strong binding only in human sperm consisted of Gal-binder (MAL II), complex-binder (PHA-L, PHA-E + L, PHA-E), Sia-binder (SNA) and one unknown specific lectin (PHA-P). The fourth cluster bound specifically with rabbit sperm consisted of GalNAc-binder (DBA, BDA and CSA).

According to the lectin binding profile based cluster (Figure 3A), the relationship of the five species were as follows: human, rabbit, boar, bull and goat, the most close species to human is rabbit, and bull and goat were close to each other. Based on previous studies [39-41], a phylogenetic tree of the five species was generated using the DNA sequence similarity (Figure 3B). Interestingly, the relationship of the five species according to the phylogenetic tree is completely consistent with that of the lectin binding based clustering. This strongly suggests that a set of selected lectins may be used for generating glycan based phylogenetic trees with high accuracy.

## Discussion

Sperm surface glycans play key roles in sperm motility, maturation and fertilization. A comprehensive profile of the sperm surface glycans will greatly facilitate both basic research (sperm glycobiology) and clinical studies, such as diagnostics of infertility. The lectin mciroarray was first introduced in 2005 [42]. Due to sensitive and specific glycan recognition of the lectins and the inherent high-throughput, and fast analysis capability of microarray, lectin microarray has already been widely applied for profiling the surface glycans of a variety of species, from bacteria to human cells [17,20,32,35]. In this study, we took advantage of a lectin microarray with 91 lectins that we have constructed previously [20], generated the lectin binding profiles for sperm of five mammals-human, boar, rabbit, bull and goat.

The lectin binding profiles showed that a variety of lectins of a wide range of glycan specificity could bind to all the five mammalian sperm. Lectins that bind to sperm of only one species were also observed. These results suggest that the majority of the surface glycan structures and composition are similar except subtle difference among the five mammalian sperm. An explanation for this phenomenon is that the five mammals are evolutionary closely related (Figure 3B). Indeed, the relationship of the five mammals according to the lectin binding profile based clustering is totally consistent with that of the phylogenetic tree [40] (Figure 3). Thus, in addition to traditional genome sequence based phylogenetic tree, lectin binding profile or glycan profile of a specific cell type, *e.g.*, sperm could also provide useful information for evolutionary studies.

The lectin binding profiles are consistent with previous studies. Many new lectins which could bind to the sperm of one species or all the 5 species were also discovered in this study. *Pisum sativum agglutinin* (PSA), a common lectin used to access acrosome reaction (AR) in fixed human sperm, AAL and UEA I, which specific bind with human sperm acrosome [43], all presented moderate bindings to the sperm on the lectin microarray, this could serve as proof of the reliability of the lectin microarray. New lectins, such as VVL, WFA, and HPA showed strong bindings to all the mammalian sperm of five species.

According to the lectin binding profiles, the level of sialic acids of non-human sperm is significantly lower than that of human, which may be related to different freezing stability of these sperm. Sialic acid, which is very abundant in glycocalyx, coating the outmost surface of sperm, is a sign of sperm maturity and protects sperm from immune recognition in the female reproductive tract [44]. Human sperm surface was rich in α2-3-(MAA and MAL II) and α2-6-(SNA and SNA I) linked sialic acids, but lack of N-acetyl and N-glycolyl neuraminic acids (LFA), which was consistent with previous reports [14,45]. Although sperm surface of other mammals are also coated with sialic acids, the abundance was significantly lower than human, especially for boar sperm. For example, cryopreservation could cause partial loss of surface sialic acids from the boar sperm [46]. It may be correlative with freeze intolerance of boar sperm and the protection of sialic acids might be able to improve the recovery rate of boar sperm, and indeed the cryoprotectors with different monosaccharides (glucose) and disaccharides (lactose, sucrose and trehalose) could significantly improve the quality of boar sperm [47,48].

Taken together, by using lectin microarray, for the first time, we have generated the lectin-sperm binding profiles of 5 mammalian species. The reliability of sperm analysis on the lectin microarray has been demonstrated by using five mammalian species. The methodology we established is generally applicable for the profiling of sperm surface glycans, thus the current lectin-sperm binding profiling could be easily expanded to other species. The current profiles provide information between 91 lectins and sperm of 5 mammalian species. To our knowledge, these are the most comprehensive lectin-sperm binding profiles. We believe that these profiles are valuable resources and references for sperm glycobiology, clinical study and also animal breeding.

## Competing interests

The authors declare that they have no competing interests.

## Authors’ contributions

AJX and LC performed the mainly experiments and drafted the manuscript. SCT, HJS and HD conceived of the study and participated in its design and coordination. AJX, PW, YHG, BW, YCW and GWC performed sperm collection and preparation. AJX, LC, YCW, SMZ and SJG involved in data analysis. All authors read and approved the final manuscript.

## Supplementary Material

Additional file 1 The classification table of lectin binding intensity of the five species sperm. Click here for file
